# Robust tracking for functional electrical stimulation cycling with unknown time-varying input delays: A switched systems approach

**DOI:** 10.3389/fnbot.2022.1022839

**Published:** 2022-10-20

**Authors:** Xianfang Tong, Yanzheng Zhu

**Affiliations:** ^1^College of Mechanical Engineering and Automation, Huaqiao University, Xiamen, China; ^2^College of Electrical Engineering and Automation, Shandong University of Science and Technology, Qingdao, China

**Keywords:** functional electrical stimulation, Lyapunov-Krasovskii functional, rehabilitation robotics, switched nonlinear system, time-varying input delay

## Abstract

Motorized functional electrical stimulation (FES) cycling has been demonstrated to have numerous health benefits for individuals suffering from neurological disorders. FES-cycling is usually designed to track the desired trajectories in real time. However, there are input delays between the exertion of the stimulation and the corresponding muscle contraction that potentially destabilize the system and undermine training efforts. Meanwhile, muscle fatigue gives rise to a time-varying input delay and decreased force. Moreover, switching between FES and motor control can be chattering and destabilizing owing to the high frequency. This article constructs Lyapunov-Krasovskii functionals to analyze the stability and robustness of the nonlinear cycling system with time-varying input delay. A new average dwell time condition is then provided to ensure the input-to-state stability of the considered systems. Finally, numerical simulations are illustrated to verify the effectiveness of the developed controller.

## 1. Introduction

Functional electrical stimulation (FES) induced cycling has shown a large number of health benefits, such as improving muscular strength (Bélanger et al., 2000), cardiovascular effects (Hooker et al., 1992), improvements in bone mineral density (Mohr et al., 1997), and so on. Therefore, FES-cycling is used for rehabilitation exercises for individuals with various neurological disorders including Parkinson's Disease, Multiple Sclerosis, traumatic brain injury, spinal cord injury, and stroke (Petrofsky, 2003; Cousin et al., 2019). It effectively improves the overall quality of life and activities of daily living of affected patients. However, there are also several challenges to the use of the closed-loop FES-cycling control approach. For instance, there are time-varying delay muscle responses to electrical stimulation (Obuz et al., 2020), unmodeled disturbances in the dynamic model, problems with founding an optimal stimulation pattern (Einar et al., 2004), and the stimulation intensity-force mapping changes as the muscle fatigues (Downey et al., 2017), etc.

An electromechanical delay (EMD) exists between the electrical stimulation input and the muscle force production, which can be modeled as an input delay (Jezernik et al., 2004; Alibeji et al., 2015; Merad et al., 2016; Obuz et al., 2020). Meanwhile, this delay is time-varying due to muscle fatigue, which results from FES eliciting, ranging from 75 to 200 ms (Ha et al., 2016). The EMD might destabilize the presented system or even degrade the performance of designed controllers (Kei et al., 2008; Alibeji et al., 2015). Therefore, the EMD presents inevitable challenges for the FES-cycling system, which can also be affected by a large number of factors, such as age (Burgess et al., 2009; Yavuz et al., 2010), gender (Yavuz et al., 2010), stimulus parameters (Downey et al., 2017), fatigue (Laura et al., 2007), and so on. Additionally, the unknown and nonlinear mapping from the constant stimulation intensity to the generated muscle force (Ding et al., 2002) can also present challenges. Hence, it must be robust to the time-varying delays and the nonlinear muscle dynamics for a closed-loop FES controller to perform the desired tracking goals. In recent decades, the EMD response of muscles has been considered when developing neuromuscular electrical stimulation (NMES)/FES controller (Sharma et al., 2011; Iasson et al., 2013; Alibeji et al., 2015; Merad et al., 2016; Allen et al., 2022). For instance, a novel predictor-type method has been developed to address the EMD in Sharma et al. (2011). However, the EMD is assumed to be constant and known to ensure uniformly ultimately bounded tracking. A globally asymptotical tracking controller is designed in Iasson et al. (2013) by assuming that there is an unknown constant delay and precise model knowledge of limb dynamics. The feedback controller is established to compensate for the known time-varying delay during isometric contractions in Merad et al. (2016). In practice, the EMD usually has an unknown time-varying delay, but it has not been fully considered to date, which is one of the main motivations for this study.

In more recent years, switched systems have received increasing attention because many practical systems can be modeled as switched systems. Motivated to improve FES-cycling performance and to complete rehabilitation tasks in different periods, a switching control strategy has been proposed in Cousin et al. (2019) and Allen et al. (2022). For instance, a cadence switching controller is employed to ensure that the motorized FES cycling system is globally exponentially stable (Cousin et al., 2019). The experiment results validate the effectiveness of the developed controller to improve the tracking performance. Similarly, a kind of robust switched controller is designed to deal with an unknown time-varying input delay in Allen et al. (2022), and semi-globally exponential tracking to an ultimate bound is guaranteed. As a popular approach, Lyapunov-Krasovskii functionals have been frequently used to analyze the stability of time-delay systems, which are very effective at deriving the stability conditions for time-delay systems. However, the stability conditions of nonlinear switched systems with time-varying delays have rarely been obtained, except for a few sporadic results (Wang et al., 2014). Hence, structuring an appropriate Lyapunov-Krasovskii functional to analyze the stability of a class of nonlinear time-varying delay switched systems, which are derived from the FES-cycling systems, is another of the main motivations of this article.

Motivated by the aforementioned discussions, a kind of tracking controller is developed for a nonlinear FES-cycling system with time-varying input delays and external disturbance in this paper. First, the mathematical model is structured using the Euler-Lagrange equations for a class of rider-tricycle systems. Based on the transferred efficiency from the muscle force to the crank, the corresponding control system is decoupled into FES and motor drive subsystems to decrease the complexity of the original nonlinear system. Then, an appropriate state-dependent switching control law is designed for these two subsystems to alleviate muscle fatigue and prolong the duration of rehabilitation training. Meanwhile, the time-varying input delay is assumed to be unknown, but it has a known upper bound. The input-to-state stability (ISS) is guaranteed for the presented FES-cycling system by using the constructed Lyapunov-Krasovskii functional, and the corresponding robust controller is designed to achieve the desired performance goal. Moreover, an average dwell time (ADT) constraint is considered between two switchings to avoid chattering induced by high frequency switching. A numerical simulation is presented to illustrate the effectiveness of the designed controller. The main contributions of this paper can be summarized as follows:

- The complex rider-tricycle system is decomposed into two subsystems by a class of switching control strategies. Meanwhile, the EMD is considered a time-varying input delay in the system.
- By constructing an appropriate Lyapunov-Krasovskii functional and introducing the ADT technique, the input-to-state stability (ISS) condition is derived for the augmented switched system with unknown time-varying input delays.
- The upper bound of time delays is only needed, which relaxes the restriction to the time-varying input delay, and hence the conservatism is decreased in this paper.

*Notation*: The notation used in this paper is fairly standard. ℝ and ℝ^+^ denote the set of real numbers and positive real numbers, respectively. The superscript *T* denotes matrix transposition, and symbol |•| denotes the Euclidean norm of a real vector. *C*^1^ stands for the space of first-order continuously differentiable functions. *I* is the identity matrix with appropriate dimensions. If |*w*|_∞_ ≤ ∞ exists, then it holds that $w\in L_{\infty }^{m}$.

## 2. Problem statement and preliminaries

**Figure 1:**
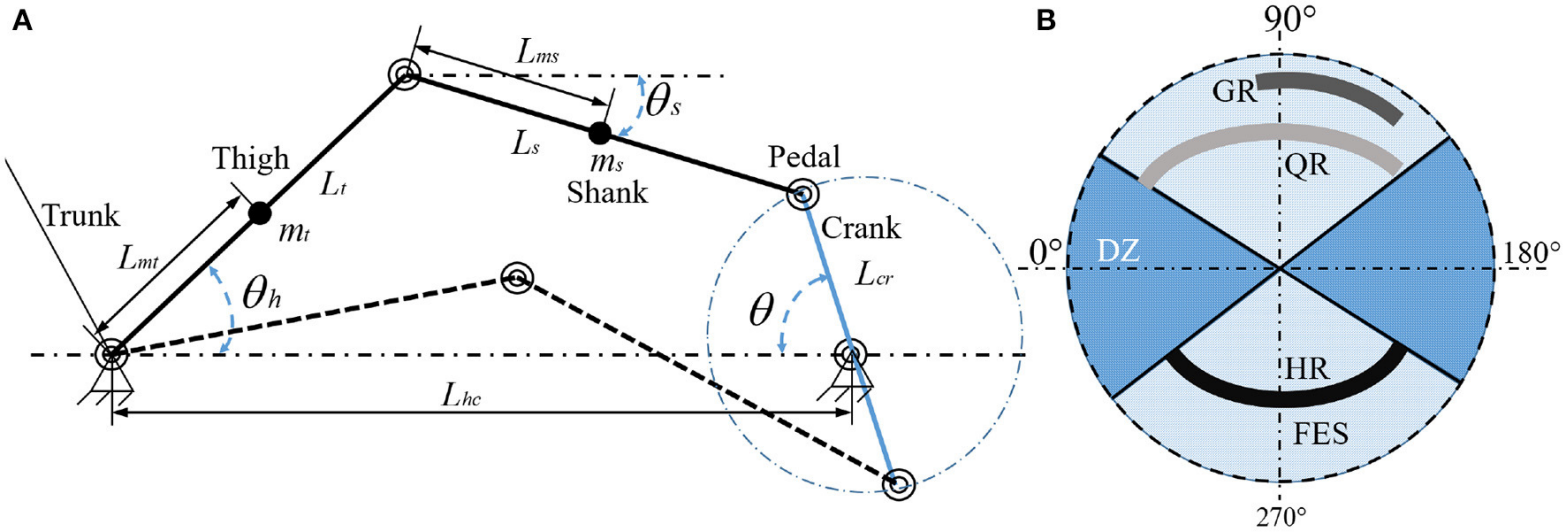
**(A)** Model of the rider-tricycle system (left). **(B)** Crank cycle pattern of FES and motor regions (right). The stimulation regions of the right leg (*GR, QR, HR*) differ 180° from the left leg (*GL, QL, HL*). Here, the stimulation regions of the left leg are omitted for simplicity.

At first, the legs of the rider are modeled as the form of the two-link. The revolute joint (hip) and the rider's feet are fixed to the cycle seat and pedals, respectively. The pedal crank arms are limited to rotate on a circle around the center of the crank with π radians. All the parameters of the rider and tricycle are transformed into those of the crank. In particular, the diagram of the presented rider-tricycle system is shown in Figure 1A. The dynamics of the rider-tricycle system can be modeled by using the Euler-Lagrange framework as used in Einar (2002):


(1) $$\begin{array}{l} M\left(\theta \right)\ddot{\theta }+C^{\prime }\left(\theta ,\dot{\theta }\right)\dot{\theta }+G\left(\theta \right)+V\left(\theta ,\dot{\theta }\right)+d\left(t\right)=\nu_{crank} \end{array}$$

where $\ddot{\theta }$ : ℝ ≥ 0 → ℝ, $\dot{\theta }$ : ℝ ≥ 0 → ℝ, θ : ℝ ≥ 0 → Θ denote the crank acceleration, cadence, and angle, respectively. The set Θ ⊆ ℝ denotes all possible crank angles. The inertial effects, centripetal-Coriolis and viscoelastic damping effects, gravitational effects, passive viscous forces, and disturbance are denoted by *M*(θ):Θ → ℝ^+^, $C^{\prime }\left(\theta ,\dot{\theta }\right):\Theta \times \mathbb{R}\to \mathbb{R}$, *G*(θ):Θ → ℝ^+^, $V\left(\theta ,\dot{\theta }\right):\Theta \times \mathbb{R}\to \mathbb{R}$, and *d*(*t*):ℝ ≥ 0 → ℝ, respectively. The crank torque (including motor and FES stimulated muscle contractions) is denoted by ν_*crank*_ ∈ ℝ, which can be defined as


(2) $$\begin{array}{l} \nu_{crank}≜\nu_{e}\left(\theta ,\dot{\theta },\tau_{e}\left(t\right),t\right)+\nu_{M}\left(\theta ,\dot{\theta },\tau_{M}\left(t\right),t\right) \end{array}$$

where $\tau_{e}\left(t\right)\in {\mathbb{R}}^{+}$ and $\tau_{M}\left(t\right)\in {\mathbb{R}}^{+}$ represent the unknown time-varying input delay of motor and FES stimulated muscle contractions, respectively. Additionally, the torques due to the motor and muscle contractions are denoted by $\nu_{e}\left(\theta ,\dot{\theta },\tau_{e}\left(t\right),t\right):\Theta \times \mathbb{R}\times {\mathbb{R}}^{+}\times \mathbb{R}\geq 0\to \mathbb{R}$ and $\nu_{M}\left(\theta ,\dot{\theta },\tau_{M}\left(t\right),t\right):\Theta \times \mathbb{R}\times {\mathbb{R}}^{+}\times \mathbb{R}\geq 0\to \mathbb{R}$, respectively, which are defined as


(3) $$\begin{array}{l} \{\begin{array}{l} \nu_{e}\left(\theta ,\dot{\theta },\tau_{e}\left(t\right),t\right)≜k_{e}\sigma_{e}\left(\theta ,\dot{\theta }\right)u\left(t-\tau_{e}\left(t\right)\right)\\ \nu_{M}\left(\theta ,\dot{\theta },\tau_{M}\left(t\right),t\right)≜\underset{m\in 𝕄}{\sum }k_{m}\sigma_{m}\left(\theta ,\dot{\theta }\right)u\left(t-\tau_{M}\left(t\right)\right) \end{array} \end{array}$$

where *u*(*t* − τ_*M*_(*t*)):ℝ × ℝ ≥ 0 → ℝ and *u*(*t* − τ_*e*_(*t*)):ℝ × ℝ ≥ 0 → ℝ stand for the FES and motor input with time-varying delays, respectively. The parameters *k*_*e*_, $k_{m}\in {\mathbb{R}}^{+},\forall m\in M$ are selectable constants, where *m* ∈ *M* ≜ {*GR, QR, HR, GL, QL, HL*} indicates gluteal (G), hamstrings (H), and quadriceps (Q) femoris muscle groups in right (R) and left (L) limbs, respectively. The right-continuous switching signals for motor and each muscle group are denoted by $\sigma_{e}\left(\theta ,\dot{\theta }\right):\Theta \times \mathbb{R}\to \left\{0,\frac{1}{2},1\right\}$ and $\sigma_{m}\left(\theta ,\dot{\theta }\right):\Theta \times \mathbb{R}\to \left\{0,\frac{1}{2},1\right\}$, respectively, which are defined as


(4) $$\begin{array}{l} \sigma_{e}\left(\theta ,\dot{\theta }\right)≜\{\begin{array}{ll} 1, & \theta \in \Theta_{\text{DZ}},t<T_{3}\\ \frac{1}{2}, & \theta \in \Theta_{\text{FES}},T_{1}<t\leq T_{2}\\ 1, & \theta \in \Theta_{\text{FES}},T_{2}<t\leq T_{3}\\ 0, & \text{otherwise} \end{array} \end{array}$$

(5) $$\begin{array}{l} \sigma_{m}\left(\theta ,\dot{\theta }\right)≜\{\begin{array}{ll} 1, & \theta \in \Theta_{\text{FES}},t\leq T_{1}\\ \frac{1}{2}, & \theta \in \Theta_{\text{FES}},T_{1}<t\leq T_{2}\\ 0, & \text{otherwise} \end{array} \end{array}$$

where Θ_FES_ denotes the union of all stimulation regions that are based on the torque transfer ratio of muscle groups and is defined as $\Theta_{\text{FES}}≜\underset{m\in 𝕄}{⋃}\left\{\Theta_{m}\right\}$. Each muscle's desired contraction region, i.e., efficient forward pedaling is denoted by Θ_*m*_, defined as Θ_*m*_ ≜ {θ ∈ Θ|*T*_*m*_(θ) > ε_*m*_}, *m* ∈ 𝕄, where *T*_*m*_(θ):Θ → ℝ and $\varepsilon_{m}\in {\mathbb{R}}^{+}$ denote a muscle contraction torque transfer ratio and a selected lower threshold, respectively. Moreover, the remainder regions (i.e., motor regions) of the crank cycle are denoted by Θ_DZ_, which is defined as Θ_DZ_ ≜ Θ/Θ_FES_. In particular, the regions of Θ_FES_ and Θ_DZ_ are illustrated in Figure 1B. For any finite time interval, it assumes that only a finite number of switches and no jump occurs in the states at a switching moment. Meanwhile, *T*_1_, *T*_2_, and *T*_3_ denote the time when the relative error first reaches *c*_1_, *c*_2_, and *c*_3_, respectively, where $c_{1}\in {\mathbb{R}}^{+}$, $c_{2}\in {\mathbb{R}}^{+}$, and $c_{3}\in {\mathbb{R}}^{+}$ are selectable constants. The parameter θ ∈ Θ_FES_, and *c*_1_ < *e*_*r*_ ≤ *c*_2_ indicates the muscle contraction torque is too small to track the desired trajectory and the torque needs to be provided by the motor. In contrast, θ ∈ Θ_FES_, *e*_*r*_ ≤ *c*_1_ indicates that the muscle contraction torque can effectively track the desired trajectory. Moreover, to avoid secondary injury during rehabilitation, the system will stop running once the relative error is greater than *c*_3_. The relative error is denoted by *e*_*r*_, which is defined as


(6) $$\begin{array}{l} e_{r}≜|\frac{x_{r}-x}{x_{r}}| \end{array}$$

where *x*_*r*_:ℝ ≥ 0 → ℝ and *x*:ℝ ≥ 0 → ℝ denote the desired and actual trajectories, respectively. Substituting Equations (2) and (3) into Equation (1) yields the switched system


(7) $$\begin{array}{l} M\left(\theta \right)\ddot{\theta }+C^{\prime }\left(\theta ,\dot{\theta }\right)\dot{\theta }+G\left(\theta \right)+V\left(\theta ,\dot{\theta }\right)+d\left(t\right)=\\ k_{e}\sigma_{e}\left(\theta ,\dot{\theta }\right)u\left(t-\tau_{e}\left(t\right)\right)+\underset{m\in 𝕄}{\sum }k_{m}\sigma_{m}\left(\theta ,\dot{\theta }\right)u\left(t-\tau_{M}\left(t\right)\right) \end{array}$$

**Remark 1**. As shown in Sharma et al. (2011), the EMD significantly increases with FES induced fatigue. Therefore, after the error reaches *c*_1_ for the moment (*T*_1_), with muscle fatigue and time delays, the muscle force will not provide enough force to track the ideal trajectory under the error *c*_1_. Then, the system will be switched to assist mode and the motor provides partial force to reduce the muscle load and the error between *T*_1_ and *T*_2_. The system will be switched to passive mode between *T*_2_ and *T*_3_, which is powered only by the motor. To avoid injury to users, the system stops running when the error reaches *c*_3_.

## 3. Control development

To facilitate controller development, we define $x_{1}≜\theta ,x_{2}≜\dot{\theta }$. After substituting them into the dynamic system (Equation 7), it obtains that


(8) $$\begin{array}{l} \{\begin{array}{l} \dot{x_{1}}=x_{2}\\ \dot{x_{2}}=-M^{-1}\left(x_{1}\right)[C^{\prime }\left(x_{1},x_{2}\right)x_{2}+G\left(x_{1}\right)+\\ \;V\left(x_{1},x_{2}\right)]-M^{-1}\left(x_{1}\right)d\left(t\right)\\ \;+M^{-1}\left(x_{1}\right)[k_{e}\sigma_{e}\left(x_{1},x_{2}\right)u\left(t-\tau_{e}\left(t\right)\right)+\\ \;\underset{m\in 𝕄}{\sum }k_{m}\sigma_{m}\left(x_{1},x_{2}\right)u\left(t-\tau_{M}\left(t\right)\right)] \end{array} \end{array}$$

Equation (7) can be rewritten as


(9) $$\begin{array}{l} \{\begin{array}{l} \dot{x}\left(t\right)=f_{\sigma \left(t\right)}\left(x,t\right)+g_{\sigma \left(t\right)}\left(x,t\right)u_{\sigma \left(t\right)}\left(t-\tau \left(t\right)\right)+w\left(t\right)\\ y\left(t\right)=x\left(t\right) \end{array} \end{array}$$

where $f_{\sigma \left(t\right)}\left(x,t\right)≜\left[\begin{array}{cc} 0 & 1\\ 0 & -M^{-1}\left(x_{1}\right)C^{\prime }\left(x_{1},x_{2}\right) \end{array}\right]x-M^{-1}\left(x_{1}\right){\left[\begin{array}{cc} 0 & G^{T}\left(x_{1}\right)+V^{T}\left(x_{1},x_{2}\right) \end{array}\right]}^{T}$,

$w\left(t\right)≜-M^{-1}\left(x_{1}\right){\left[\begin{array}{cc} 0 & d^{T}\left(t\right) \end{array}\right]}^{T}$, $g_{\sigma \left(t\right)}≜M^{-1}\left(x_{1}\right)\left[\begin{array}{cc} 0 & 0\\ k_{e}\sigma_{e}\left(x_{1},x_{2}\right) & \underset{m\in 𝕄}{\sum }k_{m}\sigma_{m}\left(x_{1},x_{2}\right) \end{array}\right]$. Moreover, *x*(*t*), $u_{\sigma \left(t\right)}\left(t-\tau \left(t\right)\right)≜{\left[\begin{array}{cc} u^{T}\left(t-\tau_{e}\left(t\right)\right) & u^{T}\left(t-\tau_{M}\left(t\right)\right) \end{array}\right]}^{T}$, and *y*(*t*) are the system state, control input, and output, respectively. The time-varying input delay τ(*t*) satisfies $\tau \left(t\right)\in \left[0,\bar{\tau }\right]$ and $\dot{\tau }<c_{k}<1$, where *c*_*k*_ is a positive constant, and the delay upper bound $\bar{\tau }$ is defined as $\bar{\tau }≜\max\left\{\tau_{e},\tau_{M}\right\}$. σ(*t*):ℝ ≥ 0 → ℜ = {σ_*e*_, σ_*m*_} is the switching signal, and associated with σ(*t*), we have the switching sequence {(σ(*t*_0_), *t*_0_), (σ(*t*_1_), *t*_1_), ..., (σ(*t*_*k*_), *t*_*k*_), ...|σ(*t*_*k*_) ∈ ℜ, *k* ∈ ℕ}, which means that the σ(*t*_*k*_)-th switching signal is active when *t* ∈ [*t*_*k*_, *t*_*k*+1_), and *k* is the switching constant. Here it supposes that only a finite number of switches can occur for any finite time interval and no jump occurs in the state within a switching instant.

The desired rider-tricycle dynamic system (system without input delay and disturbance) is defined as follows


(10) $$\begin{array}{l} \{\begin{array}{l} {\dot{x}}_{d}\left(t\right)=A_{d}x_{d}\left(t\right)+B_{d}u_{d}\left(t\right)\\ y_{d}\left(t\right)=x_{d}\left(t\right) \end{array} \end{array}$$

where *x*_*d*_(*t*), *u*_*d*_(*t*), and *y*_*d*_(*t*) are the system state, reference input, and desired output, respectively. Additionally, *A*_*d*_ (Hurwitz matrix) and *B*_*d*_ are the known constant matrices with appropriate dimensions. The state and output tracking error are defined as *r*(*t*) ≜ *x*_*d*_(*t*) − *x*(*t*) and *r*_*out*_(*t*) ≜ *y*_*d*_(*t*) − *y*(*t*), respectively. Therefore, combining Equations (9) and (10), the following augmented system can be obtained


(11) $$\begin{array}{l} \{\begin{array}{l} \dot{r}\left(t\right)={\bar{f}}_{\sigma \left(t\right)}\left(r,t\right)-g_{\sigma \left(t\right)}\left(r,t\right)u_{\sigma \left(t\right)}\left(t-\tau \left(t\right)\right)+\bar{w}\left(t\right)\\ r_{out}\left(t\right)=r\left(t\right) \end{array} \end{array}$$

where ${\bar{f}}_{\sigma \left(t\right)}\left(r,t\right)≜A_{d}r\left(t\right)-f_{\sigma \left(t\right)}\left(x,t\right)+A_{d}x\left(t\right)$ and $\bar{w}\left(t\right)≜B_{d}u_{d}\left(t\right)-w\left(t\right)$. Although the parameters of the motorized cycle-rider system shown in Equation (11) are unknown, the subsequently developed FES and motor controllers only require knowledge of the bounds about any parameter (Hooker et al., 1992).

**Definition 1:** Liberzon (2003) Let *N*_σ_(*t*_0_, *t*) denotes the number of the switching of σ(*t*) on an interval [*t*_0_, *t*). If $N_{\sigma }\left(t_{0},t\right)\leq N_{0}+\frac{t-t_{0}}{t_{\alpha }}$ holds for any *N*_0_ ≥ 0 and *t*_α_ ≥ 0, then *t*_α_ is called average dwell time.

**Definition 2:** Wang et al. (2014) A function *V*:ℝ^*n*^ × ℝ^+^ → ℝ^+^ is called uniformly proper and positive definite, if there exist functions $\underline{\alpha },\;\bar{\alpha }$ belonging to $K_{\infty }$ such that ${\underline{\alpha }}^{2}\left(\left|r\left(t\right)\right|\right)\leq V\left(r\left(t\right),t\right)\leq {\bar{\alpha }}^{2}\left(\left|r\left(t\right)\right|\right)$, ∀*r*(*t*) ∈ ℝ^*n*^, *t* ≥ 0.

**Definition 3:** Wang et al. (2014) A system is said to be input-to-state stable (ISS) if there exist functions φ, $\psi \in K_{\infty }$ and β∈KL, such that for any $w\in L_{\infty }^{m}$ and for each initial condition *r*(*t*_0_) absolutely continuous, the solution of Equation (11) exists globally and satisfies $\varphi \left(\left|r\left(t\right)\right|\right)\leq \psi \left(\underset{s\in \left[t_{0},t\right]}{\sup}w\left(s\right)\right)+\beta \left(|r\left(t_{0}\right){|}_{\bar{\tau }},t-t_{0}\right)$, 0 ≤ *t*_0_ ≤ *t*.

## 4. Stability analysis

In this section, the input-to-state stability will be investigated for the switched nonlinear system (Equation 11) under the following assumptions. According to Wang et al. (2014), the nominal system is exponentially stable and the growth restrictions on the functions are given in **Assumptions 1** and **2**, respectively.

**Assumption 1:** There exist *C*^1^ piecewise uniformly proper and positive definite functions $V_{\sigma \left(t\right)}:{\mathbb{R}}^{n}\times {\mathbb{R}}^{+}\to {\mathbb{R}}^{+}$ and $U_{\sigma \left(t\right)}:{\mathbb{R}}^{n}\times {\mathbb{R}}^{+}\to {\mathbb{R}}^{+}$ such that

(i). For each σ(*t*) ∈ ℜ, there exists a constant λ_σ(*t*)_ > 0, such that for all *t* ≥ 0


(12) $$\begin{array}{l} \frac{\partial V_{\sigma \left(t\right)}}{\partial t}\left(r,t\right)+\frac{\partial V_{\sigma \left(t\right)}}{\partial r}\left(r,t\right)\left[{\bar{f}}_{\sigma \left(t\right)}\left(r,t\right)-g_{\sigma \left(t\right)}\left(r,t\right)u_{\sigma \left(t\right)}\left(t\right)\right]\leq -\\ \lambda_{\sigma \left(t\right)}V_{\sigma \left(t\right)}\left(r,t\right). \end{array}$$

(ii). There exists a constant μ ≥ 1, such that for all σ(*t*_*i*_), σ(*t*_*j*_) ∈ ℜ and *t* ≥ 0,


(13) $$\begin{array}{l} U_{\sigma \left(t_{i}\right)}\left(r,t_{i}\right)\leq \mu U_{\sigma \left(t_{j}\right)}\left(r,t_{j}\right). \end{array}$$

**Assumption 2:** For any σ(*t*) ∈ ℜ, there exist constants *c*_*w*_ > 0, *k*_*iσ*(*t*)_ ≥ 0, (*i*=1,...,5) and $\underline{\alpha }\in K_{\infty }$, such that for all *r* ∈ ℝ^*n*^ and *t* ≥ 0, the following inequalities hold:

(i) $|\frac{\partial u_{\sigma \left(t\right)}}{\partial r}|\leq k_{1\sigma \left(t\right)}$,

(ii) $|{\bar{f}}_{\sigma \left(t\right)}\left(r,t\right){|}^{2}\leq k_{2\sigma \left(t\right)}{\underline{\alpha }}^{2}\left(\left|r\left(t\right)\right|\right)$,

(iii) ${\left(\left|g_{\sigma \left(t\right)}\left(r,t\right)||u\left(t-\tau \left(t\right)\right)\right|\right)}^{2}\leq k_{3\sigma \left(t\right)}{\underline{\alpha }}^{2}\left(\left|r\left(t\right)\right|\right)+k_{4\sigma \left(t\right)}{\underline{\alpha }}^{2}\left(\left|r\left(t-\tau \left(t\right)\right)\right|\right)$,

(iv) $|\frac{\partial V_{\sigma \left(t\right)}}{\partial r}\left(r,t\right)g_{\sigma \left(t\right)}\left(r,t\right)|\leq k_{5\sigma \left(t\right)}\underline{\alpha }\left(\left|r\left(t\right)\right|\right)$,

(v) $\left|\frac{\partial V_{\sigma \left(t\right)}}{\partial r}\left(r,t\right)\bar{w}\left(t\right)|\leq c_{w}|\bar{w}\left(t\right)\right|$.

**Theorem 1:** Under **Assumptions 1** and **2**, if there exist positive constants *c*_1σ(*t*)_ and *c*_2σ(*t*)_ such that


(14) $$\begin{array}{l} \lambda_{\sigma \left(t\right)}-\bar{\tau }k_{5\sigma \left(t\right)}^{2}-c_{1\sigma \left(t\right)}-3\bar{\tau }\left(c_{2\sigma \left(t\right)}+\frac{1}{4}k_{1\sigma \left(t\right)}^{2}\right)\left(k_{2\sigma \left(t\right)}+k_{3\sigma \left(t\right)}\right)>0 \end{array}$$

Moreover, under the ADT


(15) $$\begin{array}{l} t_{a}>\frac{\ln\;\mu }{c_{U}}, \end{array}$$

where $c_{U}≜\min\left\{\lambda_{\sigma \left(t\right)}-\bar{\tau }k_{5\sigma \left(t\right)}^{2}-c_{1\sigma \left(t\right)}-3\bar{\tau }\left(c_{2\sigma \left(t\right)}+\frac{1}{4}k_{1\sigma \left(t\right)}^{2}\right)\left(k_{2\sigma \left(t\right)}+k_{3\sigma \left(t\right)}\right),a_{\sigma \left(t\right)},b_{\sigma \left(t\right)}\right\}$, and *a*_σ(*t*)_ and *b*_σ(*t*)_ are the positive constants, the switched system (Equation 11) is ISS. Also, $\alpha^{\prime }\left(|r\left(t\right){|}_{\bar{\tau }}\right)\in K_{\infty }$ exists, meaning that the following inequality holds ${\underline{\alpha }}^{2}\left(\left|r\left(t\right)\right|\right)\leq V_{\sigma \left(t\right)}\leq U_{\sigma \left(t\right)}\left(r,t\right)\leq \alpha^{\prime }\left(|r\left(t\right){|}_{\bar{\tau }}\right)={\bar{\alpha }}^{2}\left(\left|r\left(t\right)\right|\right)+c_{1\sigma }\bar{\tau }{\underline{\alpha }}^{2}\left(|r\left(t\right){|}_{\bar{\tau }}\right)+\frac{{\bar{\tau }}^{2}}{2}c_{2\sigma }|r\left(t\right){|}_{\bar{\tau }}$, where *c*_1σ_ ≜ max{*c*_1σ(*t*)_} and *c*_2σ_ ≜ max{*c*_2σ(*t*)_}.

**Proof:** The piecewise Lyapunov-Krasovskii functional can be admitted for the system (Equation 11)


(16) $$\begin{array}{l} \begin{array}{l} U_{\sigma \left(t\right)}\left(r,t\right)=V_{\sigma \left(t\right)}\left(r,t\right)+V_{1\sigma \left(t\right)}\left(r,t\right)+V_{2\sigma \left(t\right)}\left(r,t\right) \end{array} \end{array}$$

where


(17) $$\begin{array}{l} V_{1\sigma \left(t\right)}\left(r,t\right)=c_{1\sigma \left(t\right)}\int_{t-\tau \left(t\right)}^{t}e^{a_{\sigma \left(t\right)}\left(s-t\right)}{\underline{\alpha }}^{2}\left(\left|r\left(s\right)\right|\right)ds, \end{array}$$

(18) $$\begin{array}{l} V_{2\sigma \left(t\right)}\left(r,t\right)=c_{2\sigma \left(t\right)}\int_{-\tau \left(t\right)}^{0}\int_{t+u}^{t}e^{b_{\sigma \left(t\right)}\left(s-t\right)}{\dot{r}}^{T}\left(s\right)\dot{r}\left(s\right)dsdu. \end{array}$$

Using conditions (i) and (iv) in **Assumption 2**, we achieve the following


(19) $$\begin{array}{l} \begin{array}{l} {\dot{V}}_{\sigma \left(t\right)}\left(r,t\right)=\frac{\partial V_{\sigma \left(t\right)}}{\partial t}\left(r,t\right)+\frac{\partial V_{\sigma \left(t\right)}}{\partial r}\left(r,t\right)[{\bar{f}}_{\sigma \left(t\right)}\left(r,t\right)-\\ \;g_{\sigma \left(t\right)}\left(r,t\right)u_{\sigma \left(t\right)}\left(t-\tau \left(t\right)\right)]\\ \;+\frac{\partial V_{\sigma \left(t\right)}}{\partial r}\left(r,t\right)\bar{w}\left(t\right)\\ \;\leq -\lambda_{\sigma \left(t\right)}V_{\sigma \left(t\right)}\left(r,t\right)+\frac{\partial V_{\sigma \left(t\right)}}{\partial r}\left(r,t\right)\bar{w}\left(t\right)\\ \;+k_{1\sigma \left(t\right)}|\frac{\partial V_{\sigma \left(t\right)}}{\partial r}\left(r,t\right)g_{\sigma \left(t\right)}\left(r,t\right)||\int_{t-\tau \left(t\right)}^{t}\dot{r}\left(s\right)ds|\\ \;\leq -\lambda_{\sigma \left(t\right)}V_{\sigma \left(t\right)}\left(r,t\right)+|\frac{\partial V_{\sigma \left(t\right)}}{\partial r}\left(r,t\right)\bar{w}\left(t\right)|\\ \;+\bar{\tau }|\frac{\partial V_{\sigma \left(t\right)}}{\partial r}\left(r,t\right)g_{\sigma \left(t\right)}\left(r,t\right){|}^{2}+\frac{\bar{\tau }}{4}k_{1\sigma \left(t\right)}^{2}|\dot{r}\left(t\right){|}^{2}\\ \;\leq -\lambda_{\sigma \left(t\right)}V_{\sigma \left(t\right)}\left(r,t\right)+\\ \;\bar{\tau }k_{5\sigma \left(t\right)}^{2}{\underline{\alpha }}^{2}\left(\left|r\left(t\right)\right|\right)+\frac{\bar{\tau }}{4}k_{1\sigma \left(t\right)}^{2}|\dot{r}\left(t\right){|}^{2}+c_{w}|\bar{w}\left(t\right)| \end{array} \end{array}$$

(20) $$\begin{array}{l} \begin{array}{l} {\dot{V}}_{1\sigma \left(t\right)}\left(r,t\right)=c_{1\sigma \left(t\right)}[{\underline{\alpha }}^{2}\left(\left|r\left(t\right)\right|\right)-\\ \;\left(1-\dot{\tau }\right)e^{-a_{\sigma \left(t\right)}\tau \left(t\right)}{\underline{\alpha }}^{2}\left(\left|r\left(t-\tau \left(t\right)\right)\right|\right)\\ \;+\int_{t-\tau \left(t\right)}^{t}-a_{\sigma \left(t\right)}e^{a_{\sigma \left(t\right)}\left(s-t\right)}{\underline{\alpha }}^{2}\left(\left|r\left(s\right)\right|\right)ds]\\ \;=-a_{\sigma \left(t\right)}V_{1\sigma \left(t\right)}\left(r,t\right)+c_{1\sigma \left(t\right)}[{\underline{\alpha }}^{2}\left(\left|r\left(t\right)\right|\right)\\ \;-\left(1-\dot{\tau }\right)e^{-a_{\sigma \left(t\right)}\tau \left(t\right)}{\underline{\alpha }}^{2}\left(\left|r\left(t-\tau \left(t\right)\right)\right|\right)]\\ \;\leq -a_{\sigma \left(t\right)}V_{1\sigma \left(t\right)}\left(r,t\right)+c_{1\sigma \left(t\right)}{\underline{\alpha }}^{2}\left(\left|r\left(t\right)\right|\right)\\ \;-c_{1\sigma \left(t\right)}\left(1-c_{k}\right)e^{-a_{\sigma \left(t\right)}\bar{\tau }}{\underline{\alpha }}^{2}\left(\left|r\left(t-\tau \left(t\right)\right)\right|\right) \end{array} \end{array}$$

(21) $$\begin{array}{l} \begin{array}{l} {\dot{V}}_{2\sigma \left(t\right)}\left(r,t\right)=c_{2\sigma \left(t\right)}[\dot{\tau }\int_{t-\tau \left(t\right)}^{t}e^{b_{\sigma \left(t\right)}\left(s-t\right)}{\dot{r}}^{T}\left(s\right)\dot{r}\left(s\right)ds+\\ \;\tau \left(t\right){\dot{r}}^{T}\left(t\right)\dot{r}\left(t\right)\\ \;-\int_{-\tau \left(t\right)}^{0}e^{b_{\sigma \left(t\right)}u}{\dot{r}}^{T}\left(t+u\right)\dot{r}\left(t+u\right)du\\ \;-\int_{-\tau \left(t\right)}^{0}\int_{t+u}^{t}b_{\sigma \left(t\right)}e^{b_{\sigma \left(t\right)}\left(s-t\right)}{\dot{r}}^{T}\left(s\right)\dot{r}\left(s\right)dsdu]\\ \;=c_{2\sigma \left(t\right)}[\dot{\tau }\int_{t-\tau \left(t\right)}^{t}e^{b_{\sigma \left(t\right)}\left(s-t\right)}{\dot{r}}^{T}\left(s\right)\dot{r}\left(s\right)ds+\\ \;\tau \left(t\right){\dot{r}}^{T}\left(t\right)\dot{r}\left(t\right)\\ \;-\int_{t-\tau \left(t\right)}^{t}e^{b_{\sigma \left(t\right)}\left(s-t\right)}{\dot{r}}^{T}\left(s\right)\dot{r}\left(s\right)ds]-b_{\sigma \left(t\right)}V_{2\sigma \left(t\right)}\left(r,t\right)\\ \;\leq c_{2\sigma \left(t\right)}[\tau \left(t\right){\dot{r}}^{T}\left(t\right)\dot{r}\left(t\right)-\\ \;\left(1-c_{k}\right)\int_{t-\tau }^{t}e^{b_{\sigma \left(t\right)}\left(s-t\right)}{\dot{r}}^{T}\left(s\right)\\ \;\dot{r}\left(s\right)ds]-b_{\sigma \left(t\right)}V_{2\sigma \left(t\right)}\left(r,t\right)\\ \;\leq c_{2\sigma \left(t\right)}\bar{\tau }|\dot{r}\left(t\right){|}^{2}-b_{\sigma \left(t\right)}V_{2\sigma \left(t\right)}\left(r,t\right) \end{array} \end{array}$$

(22) $$\begin{array}{l} \begin{array}{l} {\dot{U}}_{\sigma \left(t\right)}\left(r,t\right)\leq -\lambda_{\sigma \left(t\right)}V_{\sigma \left(t\right)}\left(r,t\right)-a_{\sigma \left(t\right)}V_{1\sigma \left(t\right)}\left(r,t\right)-b_{\sigma \left(t\right)}\\ \;V_{2\sigma \left(t\right)}\left(r,t\right)\\ \;+\left(\bar{\tau }k_{5\sigma \left(t\right)}^{2}+c_{1\sigma \left(t\right)}\right){\underline{\alpha }}^{2}\left(\left|r\left(t\right)\right|\right)+\left(c_{2\sigma \left(t\right)}+\frac{1}{4}k_{1\sigma \left(t\right)}^{2}\right)\\ \;\bar{\tau }|\dot{r}\left(t\right){|}^{2}\\ \;-c_{1\sigma \left(t\right)}\left(1-c_{k}\right)e^{-a_{\sigma \left(t\right)}\bar{\tau }}{\underline{\alpha }}^{2}\left(\left|r\left(t-\tau \left(t\right)\right)\right|\right)+c_{w}|\bar{w}\left(t\right)| \end{array} \end{array}$$

Using


(23) $$\begin{array}{l} |\dot{r}\left(t\right){|}^{2}\leq 3|{\bar{f}}_{\sigma \left(t\right)}\left(r,t\right){|}^{2}+3|g_{\sigma \left(t\right)}\left(r,t\right)u_{\sigma \left(t\right)}\left(t\right){|}^{2}+3|\bar{w}\left(t\right){|}^{2} \end{array}$$

and conditions (ii) and (iii) in **Assumption 2**, we obtain


(24) $$\begin{array}{l} |\dot{r}\left(t\right){|}^{2}\leq 3k_{2\sigma \left(t\right)}{\underline{\alpha }}^{2}\left(\left|r\left(t\right)\right|\right)+3k_{3\sigma \left(t\right)}{\underline{\alpha }}^{2}\left(\left|r\left(t\right)\right|\right)+3|\bar{w}\left(t\right){|}^{2} \end{array}$$

Then, combining Equations (22) and (24), it deduces that


(25) $$\begin{array}{l} \begin{array}{l} {\dot{U}}_{\sigma \left(t\right)}\left(r,t\right)\leq -\lambda_{\sigma \left(t\right)}V_{\sigma \left(t\right)}\left(r,t\right)-a_{\sigma \left(t\right)}V_{1\sigma \left(t\right)}\left(r,t\right)\\ \;-b_{\sigma \left(t\right)}V_{2\sigma \left(t\right)}\left(r,t\right)\\ \;+[\bar{\tau }k_{5\sigma \left(t\right)}^{2}+c_{1\sigma \left(t\right)}+3\bar{\tau }\left(c_{2\sigma \left(t\right)}+\frac{1}{4}k_{1\sigma \left(t\right)}^{2}\right)\\ \;\left(k_{2\sigma \left(t\right)}+k_{3\sigma \left(t\right)}\right)]{\underline{\alpha }}^{2}\left(\left|r\left(t\right)\right|\right)\\ \;-c_{1\sigma \left(t\right)}\left(1-c_{k}\right)e^{-a_{\sigma \left(t\right)}\bar{\tau }}{\underline{\alpha }}^{2}\left(\left|r\left(t-\tau \left(t\right)\right)\right|\right)+w^{\prime }\left(t\right)\\ \;\leq -[\lambda_{\sigma \left(t\right)}-\bar{\tau }k_{5\sigma \left(t\right)}^{2}-c_{1\sigma \left(t\right)}-3\bar{\tau }\left(c_{2\sigma \left(t\right)}+\frac{1}{4}k_{1\sigma \left(t\right)}^{2}\right)\\ \;\left(k_{2\sigma \left(t\right)}+k_{3\sigma \left(t\right)}\right)]V_{\sigma \left(t\right)}\left(r,t\right)\\ \;-a_{\sigma \left(t\right)}V_{1\sigma \left(t\right)}\left(r,t\right)-b_{\sigma \left(t\right)}V_{2\sigma \left(t\right)}\left(r,t\right)+w^{\prime }\left(t\right)\\ \;\leq -c_{U}U_{\sigma \left(t\right)}\left(r,t\right)+w^{\prime }\left(t\right) \end{array} \end{array}$$

where $w^{\prime }\left(t\right)≜c_{w}|\bar{w}\left(t\right)|+3\bar{\tau }\left(c_{2\sigma \left(t\right)}+\frac{1}{4}k_{1\sigma \left(t\right)}^{2}\right)|\bar{w}\left(t\right){|}^{2}$.

Integrating the inequality (Equation 13), it holds that


(26) $$\begin{array}{l} U_{\sigma \left(t\right)}\left(r,t\right)\leq e^{-c_{U}\left(t-t_{k}\right)}U_{\sigma \left(t_{k}\right)}\left(r,t_{k}\right)+\int_{t_{k}}^{t}e^{-c_{U}\left(t-s\right)}w^{\prime }\left(s\right)ds \end{array}$$

For the interval [*t*_0_, *t*) that contains *N*_σ_(*t*_0_, *t*) + 1 switchings and $t_{N_{\sigma }}=t^{-}$, it derives that (*N*_σ_(*t*_0_, *t*) = *N*_σ_)


(27) $$\begin{array}{l} \begin{array}{l} U_{\sigma \left(t\right)}\left(r,t\right)\leq \mu e^{-c_{U}\left(t-t_{N_{\sigma }}\right)}U_{\sigma \left(t_{N_{\sigma }}\right)}\left(r,t_{N_{\sigma }}\right)+\\ \;\mu \int_{t_{N_{\sigma }}}^{t}e^{-c_{U}\left(t-s\right)}w^{\prime }\left(s\right)ds\\ \;\leq \mu e^{-c_{U}\left(t-t_{N_{\sigma }}\right)}U_{\sigma \left(t_{N_{\sigma }-1}\right)}\left(r,t_{N_{\sigma }-1}\right)+\\ \;\mu \int_{t_{N_{\sigma }}}^{t}e^{-c_{U}\left(t-s\right)}w^{\prime }\left(s\right)ds\\ \;+\mu e^{-c_{U}\left(t-t_{N_{\sigma }}\right)}\int_{t_{N_{\sigma }-1}}^{t_{N_{\sigma }}}{\dot{U}}_{\sigma \left(s\right)}\left(r,s\right)ds\\ \;\leq \mu e^{-c_{U}\left(t-t_{N_{\sigma }}\right)}U_{\sigma \left(t_{N_{\sigma }-1}\right)}\left(r,t_{N_{\sigma }-1}\right)+\\ \;\mu \int_{t_{N_{\sigma }}}^{t}e^{-c_{U}\left(t-s\right)}w^{\prime }\left(s\right)ds\\ \;+\mu e^{-c_{U}\left(t-t_{N_{\sigma }}\right)}\int_{t_{N_{\sigma }-1}}^{t_{N_{\sigma }}}-c_{U}U_{\sigma \left(s\right)}\left(r,s\right)ds+\\ \;\mu \int_{t_{N_{\sigma }-1}}^{t_{N_{\sigma }}}e^{-c_{U}\left(t-s\right)}w^{\prime }\left(s\right)ds\\ \;\leq \mu e^{-c_{U}\left(t-t_{N_{\sigma }-1}\right)}U_{\sigma \left(t_{N_{\sigma }-1}\right)}\left(r,t_{N_{\sigma }-1}\right)+\\ \;\mu \int_{t_{N_{\sigma }-1}}^{t}e^{-c_{U}\left(t-s\right)}w^{\prime }\left(s\right)ds\\ \;\vdots \\ \;\leq \mu^{N_{\sigma }}e^{-c_{U}\left(t-t_{0}\right)}U_{\sigma \left(t_{0}\right)}\left(r,t_{0}\right)+\\ \;\mu^{N_{\sigma }}\int_{t_{0}}^{t}e^{-c_{U}\left(t-s\right)}w^{\prime }\left(s\right)ds \end{array} \end{array}$$

Using the ADT definition, assuming that $\frac{\ln\;\mu }{t_{a}}-c_{U}<0$, it yields that


(28) $$\begin{array}{l} \begin{array}{l} U_{\sigma \left(t\right)}\left(r,t\right)\leq \mu^{N_{\sigma }}e^{-c_{U}\left(t-t_{0}\right)}U_{\sigma \left(t_{0}\right)}\left(r,t_{0}\right)+\\ \;\mu^{N_{\sigma }}\int_{t_{0}}^{t}e^{-c_{U}\left(t-s\right)}w^{\prime }\left(s\right)ds\\ \;\leq \mu^{N_{0}}e^{\left(-c_{U}+\frac{\ln\;u}{t_{a}}\right)\left(t-t_{0}\right)}U_{\sigma \left(t_{0}\right)}\left(r,t_{0}\right)+\\ \;\mu^{N_{0}}\int_{t_{0}}^{t}e^{\left(-c_{U}+\frac{\ln\;u}{t_{a}}\right)\left(t-s\right)}w^{\prime }\left(s\right)ds\\ \;\leq \mu^{N_{0}}e^{\left(-c_{U}+\frac{\ln\;u}{t_{a}}\right)\left(t-t_{0}\right)}U_{\sigma \left(t_{0}\right)}\left(r,t_{0}\right)+\\ \;\mu^{N_{0}}\frac{t_{a}}{t_{a}c_{U}-\ln\mu }\underset{s\in \left[t_{0},t\right]}{\sup}w^{\prime }\left(s\right) \end{array} \end{array}$$

From Equations (16) to (18), it can be shown $V_{1\sigma \left(t\right)}\left(r,t\right)\leq c_{1\sigma }\bar{\tau }{\underline{\alpha }}^{2}\left(|r\left(t\right){|}_{\bar{\tau }}\right)$, $V_{2\sigma \left(t\right)}\left(r,t\right)\leq \frac{{\bar{\tau }}^{2}}{2}c_{2\sigma }|r\left(t\right){|}_{\bar{\tau }}$, therefore, the following inequality holds


(29) $$\begin{array}{l} {\underline{\alpha }}^{2}\left(\left|r\left(t\right)\right|\right)\leq V_{\sigma \left(t\right)}\leq U_{\sigma \left(t\right)}\left(r,t\right)\leq \alpha^{\prime }\left(|r\left(t\right){|}_{\bar{\tau }}\right) \end{array}$$

By Equations (28) and (29), it follows that


(30) $$\begin{array}{l} {\underline{\alpha }}^{2}\left(\left|r\left(t\right)\right|\right)\leq U_{\sigma \left(t\right)}\left(r,t\right)\leq \alpha^{\prime }\left(|r\left(t_{0}\right){|}_{\bar{\tau }}\right)\mu^{N_{0}}e^{\left(-c_{U}+\frac{\ln\;\mu }{t_{a}}\right)\left(t-t_{0}\right)}+\\ \;\mu^{N_{0}}\frac{t_{a}}{t_{a}c_{U}-\ln\;\mu }\underset{s\in \left[t_{0},t\right]}{\sup}w^{\prime }\left(s\right) \end{array}$$

As we know ${\underline{\alpha }}^{2}$(|*r*(*t*)|) belongs to $K_{\infty }$ and it is apparent that $\alpha^{\prime }\left(|r\left(t_{0}\right){|}_{\bar{\tau }}\right)\mu^{N_{0}}e^{\left(-c_{U}+\frac{\ln\;\mu }{t_{a}}\right)\left(t-t_{0}\right)}$ and $\mu^{N_{0}}\frac{t_{a}}{t_{a}c_{U}-\ln\;\mu }\underset{s\in \left[t_{0},t\right]}{\sup}w^{\prime }\left(s\right)$ belong to KL and $K_{\infty }$, respectively. Therefore, the system (Equation 11) is ISS. The proof is completed.

## 5. Controller design

To ensure the ISS of the presented system, we need to design an appropriate control law that meets condition (i) of **Assumption 1**. Therefore, a controller that satisfies this condition is designed in this section. The state feedback controller is developed as follows


(31) $$\begin{array}{l} u_{\sigma \left(t\right)}=K_{r\sigma \left(t\right)}\left(x_{r}\left(t\right)-x\left(t\right)\right)=K_{r\sigma \left(t\right)}r\left(t\right) \end{array}$$

where *K*_*rσ*(*t*)_ is the controller gain matrix to be designed.

**Theorem 2:** Consider the switched delay error system (Equation 11) and condition (i) of **Assumption 1**. The controller (Equation 31) can guarantee system (Equation 11) is ISS, if there exists positive matrix *K*_*rσ*(*t*)_ such that $\left(\lambda_{\sigma \left(t\right)}+\frac{k_{2\sigma \left(t\right)}+1}{2}\right)I\leq g_{\sigma \left(t\right)}K_{r\sigma \left(t\right)}$.

**Proof:** To guarantee ISS for switched system (Equation 11), the designed controller (Equation 31) needs to satisfy condition (i) of **Assumption 1** for the nominal system (without input delays and external disturbance). The piecewise Lyapunov function candidate is selected as $V_{\sigma \left(t\right)}\left(r,t\right)=\frac{1}{2}r^{T}\left(t\right)r\left(t\right)$. Then, its time derivative of *V*_σ(*t*)_(*r, t*) is obtained in the following:


(32) $$\begin{array}{l} \begin{array}{l} {\dot{V}}_{\sigma \left(t\right)}\left(r,t\right)=r^{T}\left(t\right)\dot{r}\left(t\right)\\ \;=r^{T}\left(t\right)\left({\bar{f}}_{\sigma \left(t\right)}\left(r,t\right)-g_{\sigma \left(t\right)}\left(r,t\right)u_{\sigma \left(t\right)}\right)\\ \;=r^{T}\left(t\right){\bar{f}}_{\sigma \left(t\right)}\left(r,t\right)-r^{T}\left(t\right)g_{\sigma \left(t\right)}\left(r,t\right)K_{r\sigma \left(t\right)}r\left(t\right)\\ \;\leq \frac{1}{2}r^{T}\left(t\right)r\left(t\right)+\frac{1}{2}{\bar{f}}_{\sigma \left(t\right)}^{2}\left(r,t\right)+r^{T}\left(t\right)(-g_{\sigma \left(t\right)}\left(r,t\right)\\ \;K_{r\sigma \left(t\right)})r\left(t\right)\\ \;\leq \frac{k_{2\sigma \left(t\right)}}{2}{\underline{\alpha }}^{2}\left(\left|r\left(t\right)\right|\right)-\left(\lambda_{\sigma \left(t\right)}+\frac{k_{2\sigma \left(t\right)}}{2}\right)r^{T}\left(t\right)r\left(t\right)\\ \;+r^{T}\left(t\right)\left(\left(\lambda_{\sigma \left(t\right)}+\frac{k_{2\sigma \left(t\right)}+1}{2}\right)I-g_{\sigma \left(t\right)}K_{r\sigma \left(t\right)}\right)r\left(t\right)\\ \;\leq -\lambda_{\sigma \left(t\right)}V_{\sigma \left(t\right)}\left(r,t\right)+r^{T}\left(t\right)[\left(\lambda_{\sigma \left(t\right)}+\frac{k_{2\sigma \left(t\right)}+1}{2}\right)\\ \;I-g_{\sigma \left(t\right)}K_{r\sigma \left(t\right)}]r\left(t\right) \end{array} \end{array}$$

where λ_σ(*t*)_ > 0. Moreover, λ_σ(*t*)_ and *K*_*rσ*(*t*)_ satisfy $\left(\lambda_{\sigma \left(t\right)}+\frac{k_{2\sigma \left(t\right)}+1}{2}\right)I\leq g_{\sigma \left(t\right)}K_{r\sigma \left(t\right)}$. The proof is completed.

**Remark 2**. Considering Equation (19), in order to guarantee input-to-state stability, condition (i) of **Assumption 1** must be satisfied via designing an appropriate control law. It means that any controller of the nominal system (without input delays and external disturbance) fulfills **Theorem 2**.

## 6. Illustrative example

In this section, a practical example is presented to illustrate the effectiveness of the proposed method. The parameters of the rider-tricycle system are listed at Table 1. The following equations are established based on Figure 1A:


(33) $$\begin{array}{l} \{\begin{array}{l} L_{t}\cos\theta_{h}+L_{s}\cos\theta_{s}+L_{cr}\cos\theta =L_{hc}\\ L_{t}\sin\theta_{h}+L_{s}\sin\theta_{s}-L_{cr}\sin\theta =0 \end{array} \end{array}$$

**Table 1:** Parameters of the rider-tricycle model (Einar et al., 2010).

Length of thigh	*L*_*t*_ = 0.42 m	Mass of thigh	*m*_*t*_ = 10.0 kg
Length of shank	*L*_*s*_ = 0.50 m	Mass of shank	*m*_*s*_ = 3.5 kg
Length of crank	*L*_*cr*_ = 0.12 m	Length from hip to thigh center of mass	*L*_*mt*_ = 0.244 m
Length from hip to center of crank	*L*_*hc*_ = 0.69 m	Length from knee to shank center of mass	*L*_*ms*_ = 0.279 m
Thigh moment of inertia	$I_{t}=2,431\;\text{kg}\cdot {\text{cm}}^{2}$	Shank moment of inertia	$I_{s}=476\text{kg}\cdot {\text{cm}}^{2}$
The inertia of the tricycle	$M_{cycle}=1,500\;\text{kg}\cdot {\text{cm}}^{2}$		

The above equations can be solved based on the parameters provided at Table 1. Hence, the expression for angles θ_*h*_ and θ_*s*_ can be expressed by θ to reduce the degree-of-freedoms and dimensions of the rider-tricycle system.

Next, using the Euler-Lagrange equation, the inertia *M*_*m*_(θ), centripetal-Coriolis *C*(θ), and gravitational effects *G*_*m*_(θ) (right or left lag) are respectively expressed with respect to the crank angle as follows:


$$\begin{array}{c} M_{m}\left(\theta \right)=\left(m_{t}L_{mt}^{2}+m_{s}L_{t}^{2}+I_{t}\right){\left(\frac{d\theta_{h}}{d\theta }\right)}^{2}+2m_{s}L_{t}L_{ms}\cos\left(\theta_{h}-\theta_{s}\right)\\ \frac{d\theta_{h}}{d\theta }\frac{d\theta_{s}}{d\theta }+\left(m_{s}L_{ms}^{2}+I_{s}\right){\left(\frac{d\theta_{s}}{d\theta }\right)}^{2}, \end{array}$$

$$\begin{array}{l} \begin{array}{l} C\left(\theta \right)=\left(m_{t}L_{mt}^{2}+m_{s}L_{t}^{2}+I_{t}\right)\frac{d\theta_{h}}{d\theta }\frac{d^{2}\theta_{h}}{d\theta^{2}}+m_{s}L_{t}L_{ms}\cos\left(\theta_{h}-\theta_{s}\right)\\ \;\left(\frac{d^{2}\theta_{h}}{d\theta^{2}}\frac{d\theta_{s}}{d\theta }+\frac{d\theta_{h}}{d\theta }\frac{d^{2}\theta_{s}}{d\theta^{2}}\right)\\ \;+\left(m_{s}L_{ms}^{2}+I_{s}\right)\frac{d\theta_{s}}{d\theta }\frac{d^{2}\theta_{s}}{d\theta^{2}}-m_{s}L_{t}L_{ms}\\ \;\left(\frac{d\theta_{h}}{d\theta }-\frac{d\theta_{s}}{d\theta }\right)\frac{d\theta_{h}}{d\theta }\frac{d\theta_{s}}{d\theta }\sin\left(\theta_{h}-\theta_{s}\right), \end{array} \end{array}$$

$$\begin{array}{l} G_{m}\left(\theta \right)=-g\left[\left(m_{t}L_{mt}+m_{s}L_{t}\right)\cos\left(\theta_{h}\right)\frac{d\theta_{h}}{d\theta }+m_{s}L_{ms}\cos\left(\theta_{s}\right)\frac{d\theta_{s}}{d\theta }\right], \end{array}$$

we obtain


(34) $$\begin{array}{l} M\left(\theta \right)=M_{m}\left(\theta \right)+M_{m}\left(\theta +\pi \right)+M_{cycle}, \end{array}$$

(35) $$\begin{array}{l} C^{\prime }\left(\theta \right)=C\left(\theta \right)+C\left(\theta +\pi \right), \end{array}$$

(36) $$\begin{array}{l} G\left(\theta \right)=G_{m}\left(\theta \right)+G_{m}\left(\theta +\pi \right). \end{array}$$

Additionally, the passive viscous forces and external disturbance are applied as $V\left(\dot{\theta }\right)=0.1\dot{\theta }$ and *d*(*t*) = 0.1sin(0.25*t*), respectively. The desired crank position θ_*d*_ and velocity ${\dot{\theta }}_{d}$ are designed as


(37) $$\begin{array}{l} \theta_{d}\left(t\right)=\frac{5\pi }{3}t-\frac{5}{2}{\dot{\theta }}_{d} \end{array}$$

(38) $$\begin{array}{l} {\dot{\theta }}_{d}\left(t\right)=\frac{5\pi }{3}\left[1-e^{-\frac{2}{5}t}\right], \end{array}$$

Moreover, we define *x*_*d*1_ ≜ θ_*d*_ and $x_{d2}≜{\dot{\theta }}_{d}$, then we have


(39) $$\begin{array}{l} \left[\begin{array}{c} {\dot{x}}_{d1}\\ {\dot{x}}_{d2} \end{array}\right]=\left[\begin{array}{cc} 0 & 1\\ 0 & -\frac{2}{5} \end{array}\right]\left[\begin{array}{c} x_{d1}\\ x_{d2} \end{array}\right]+\left[\begin{array}{c} 0\\ \frac{2\pi }{3} \end{array}\right] \end{array}$$

Next, the system (Equation 11) can be obtained. The initial conditions for the system are selected as *x*_1_ = 1 and *x*_2_ = 0. With the duration of FES, the time delay of muscle response will be increasing. Thus, the motor and muscle input delay are assumed as τ_*e*_ = 5 ms and $\tau_{m}=250e^{\left(t-200\right)/40}$ ms. We obtain *c*_*k*_ = 0.00625 and $\bar{\tau }=250$ ms. Choosing the Lyapunov functions $V_{1}\left(r,t\right)=r_{1}^{2}\left(t\right)+r_{2}^{2}\left(t\right)$ and $V_{2}\left(r,t\right)=r_{1}^{2}\left(t\right)+r_{2}^{2}\left(t\right)$, we obtain μ ≥ 1.

Given *k*_*e*_ = 2, *k*_*m*_ = 2|sin(*x*_1_)|, *c*_1_ = 0.05, *c*_2_ = 0.1, *c*_3_ = 0.15, $c_{1\sigma \left(t\right)}=\frac{1}{2}$, $c_{2\sigma \left(t\right)}=\frac{1}{4}$, *a*_σ(*t*)_ = 1, $b_{\sigma \left(t\right)}=\frac{1}{2}$, and μ = 1.1, we derive λ = 55, *t*_*a*_ = 0.19s, and *K*_*rσ*(*t*)_ = 7*I*.

**Figure 2:**
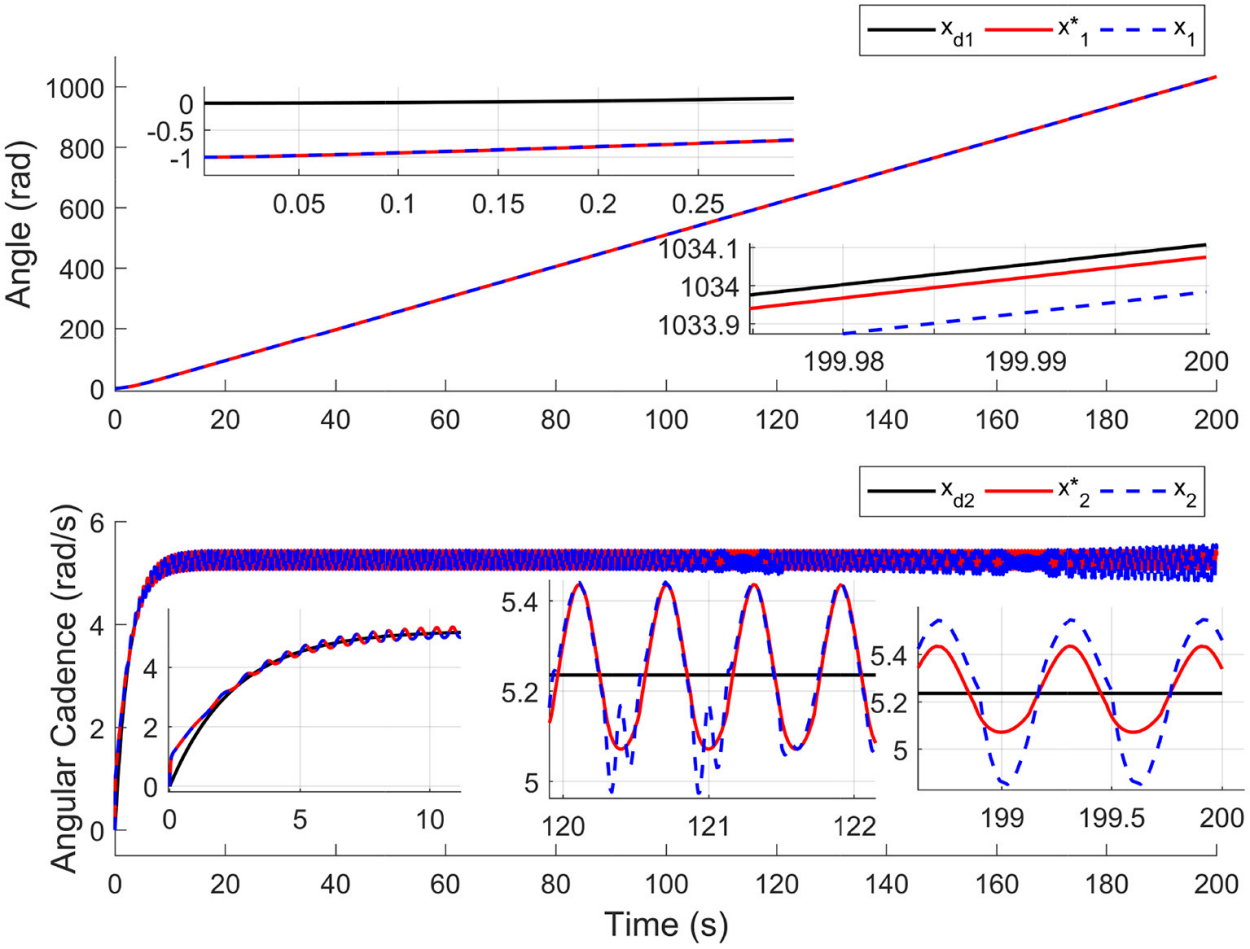
The tracking performance for nominal system and time delays system. The black solid lines (*x*_*d*1_, *x*_*d*2_), the red solid lines ($x_{1}^{*},x_{2}^{*}$), and the blue dotted lines (*x*_1_, *x*_2_) represent the trajectories of target, nominal system, and time delay system, respectively.

**Figure 3:**
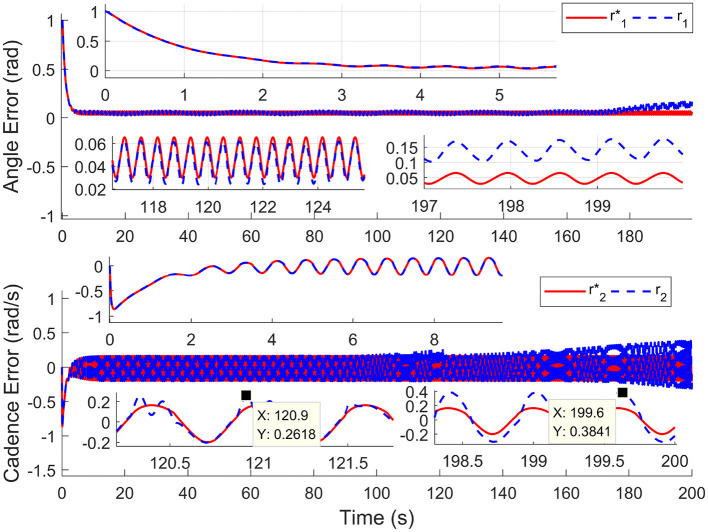
The tracking errors for nominal system and time delays system. The red solid lines ($r_{1}^{*},r_{2}^{*}$) and the blue dotted lines (*r*_1_, *r*_2_) represent the tracking errors of the nominal and time delay systems, respectively.

**Figure 4:**
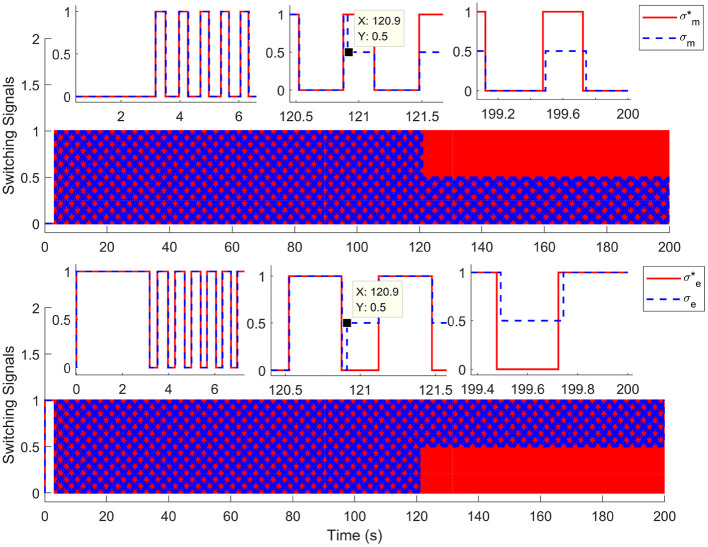
The switching signals for nominal system and time delays system. The red solid lines ($\sigma_{m}^{*},\sigma_{e}^{*}$) and the blue dotted lines (σ_*m*_, σ_*e*_) represent the switching signals of the nominal and time delay systems, respectively.

For comparison, the simulations are completed using the nominal system (without input delay and external disturbance) and time-varying input delays, which range from 1.7 to 250 ms. Results in Figure 2 depict the tracking trajectories of the nominal system and time delay system. Results in Figure 3 depict the tracking errors of nominal and time delay systems. It shows that, as the input delay is increasing, the proposed controller can ensure the boundedness of the tracking errors. The cadence error reaches 5% (0.261799rad/s) for the first time at 120.9 s. Whereafter, the assist mode is activated. The maximum cadence error of the delay system is 7.3% (0.3841rad/s). Correspondingly, the maximum cadence error of the nominal system is 3.8% (0.1998rad/s), as the cadence reaches 80% of the expected working aim. The results in Figure 4 depict the switching signals of the nominal and time delay systems. At the beginning of the simulation, the system is not switched to the FES subsystem until the cadence reaches 80% of the ideal cadence.

## 7. Conclusion

In this paper, a model for FES-cycling with motor driven assistance is presented, which contains the effects of time-varying input delay and unknown disturbances. The input-to-state stability of the switched nonlinear FES-cycling system with time delays and disturbances has been analyzed. The state-dependent switching laws are considered for the switched nonlinear rider-tricycle system. Moreover, the property of ADT switching is introduced to avoid the frequent switching and chattering of subsystems. Novel ISS criteria have been derived by constructing an appropriate Lyapunov-Krasovskii functional under the ADT constraint. Compared with the existing results, the input delay in this paper is not assumed to be known but only requires its upper bound. Simulation results indicate the performance of the controller over a scope of time-varying input delays and the robustness to time-varying input delays up to 250 ms. In future work, the master-slave synchronous control for FES-cycling with electromechanical delays is an interesting topic (Liu et al., 2022; Shi et al., 2022).

## Data availability statement

The original contributions presented in the study are included in the article/supplementary material, further inquiries can be directed to the corresponding author/s.

## Author contributions

YZ provides substantial contributions to the conception or design of the work and approval for publication of the content. XT drafts the work critically for important intellectual content. Both authors contributed to the article and approved the submitted version.

## Funding

This work was supported in part by the National Natural Science Foundation of China (under Grant Nos. 62222310 and 61973131) and in part by the Fujian Outstanding Youth Science Fund under Grant No. 2020J06022.

## Conflict of interest

The authors declare that the research was conducted in the absence of any commercial or financial relationships that could be construed as a potential conflict of interest.

## Publisher's note

All claims expressed in this article are solely those of the authors and do not necessarily represent those of their affiliated organizations, or those of the publisher, the editors and the reviewers. Any product that may be evaluated in this article, or claim that may be made by its manufacturer, is not guaranteed or endorsed by the publisher.
